# *Stenotrophomonas maltophilia*: Genotypic Characterization of Virulence Genes and The Effect of Ascorbic Acid on Biofilm Formation

**DOI:** 10.1007/s00284-022-02869-7

**Published:** 2022-05-05

**Authors:** Amira ElBaradei, Marwa Atef Yakout

**Affiliations:** 1 Department of Microbiology and Immunology, Faculty of Pharmacy, Pharos University in Alexandria, Alexandria, Egypt; 2 Alexandria University Hospital, Alexandria University, Alexandria, Egypt

## Abstract

*Stenotrophomonas maltophilia* is an environmental bacterium that has gained a lot of attention, as a nosocomial pathogen associated with significant mortality rates. Biofilm formation is considered the corner stone for establishing infections in many bacteria including *S. maltophilia.* The aim of this study was the genotypic characterization of the different virulence-associated genes and the investigation of the effect of ascorbic acid on *S. maltophilia* biofilm formation. A total of 20 *S. maltophilia* isolates from different sources were included in this study. Genes encoding different virulence factors were investigated genotypically. These included *stmPr1*, *stmPr2*, s*mlt3773 locus*, *smf-1*, *rpfF*, *rmlA* and *spgM*. Biofilm formation was investigated phenotypically. The effect of ascorbic acid on biofilm formation was investigated using MIC as well as sub-inhibitory concentrations. Many of the isolates harbored both serine proteases genes *stmPr-1* and *stmPr-2*. Fourteen (70%) of the 20 isolates carried *stmPr-1* and 15 (75%) had *stmPr-2*. Most of the isolates (95%) possessed *smlt-3773 locus*. Genes linked to biofilm formation such as *smf-1*, *rpfF*, *rmlA* and *spgM*, were found in (90%), (45%), (85%) and (30%) of the isolates, respectively. Phenotypically, all *S. maltophilia* isolates (100%) were biofilm producers. Fifteen (75%) were strong biofilm producers and 5 (25%) were moderate biofilm producers. In attempts to seek a non-chemotherapeutic alternative that can hinder biofilm formation without provoking antimicrobial resistance, the results, herein, showed that ascorbic acid inhibits biofilm formation in a dose-dependent manner.

**Supplementary Information:**

The online version contains supplementary material available at 10.1007/s00284-022-02869-7.

## Introduction

*Stenotrophomonas maltophilia* is a non-fermenting, Gram-negative bacilli. It is an obligate aerobe that is motile with polar flagella [1]. Originally an environmental bacterium, it has gained a lot of attention as a nosocomial pathogen associated with significant mortality rates [2]. However, reports of cases of community acquired infections have also been implicated [1].

Although not abundantly virulent, *S. maltophilia* possess various virulence-associated factors and it is able to persist on different surfaces due to biofilm formation [2]. These virulence factors include extracellular proteases and esterase [2]. There are two important serine proteases StmPr1 and StmPr2 both of which are substrates for Xps, a type II secretion system (T2SS) [3]. Other virulence factors include fimbriae which are crucial for adherence to surfaces and biofilm formation [4]. Moreover, extracellular polysaccharides (EPS) are essential for biofilm structure. In fact, biofilms are held by these polysaccharides to form multi-layered well-organized structures [5].

*Stenotrophomonas maltophilia* biofilms are considered a key player in the bacterium ability to cause diseases [6]. Biofilms act as a shield for bacteria within, they guard the bacteria from antibacterial agents and the host immune responses [7]. Moreover, they allow these bacteria to persist and colonize different surfaces including the surfaces of medical equipment, from which they are very challenging to remove [6]. This paves the way for *S. maltophilia* to establish different infections especially in hospital settings [7].

To overcome this challenge, many agents have been investigated to reduce biofilm formation, to achieve utmost clinical outcomes [2]. Vitamin C (ascorbic acid) has been shown to disrupt biofilm formation in some bacterial species [8, 9]. However, to the best of our knowledge its effect on *S. maltophilia* biofilms has not been investigated, yet. Hence, the aim of this study was the genotypic characterization of the different virulence-associated genes and the investigation of the effect of ascorbic acid on *S. maltophilia* biofilm formation.

## Materials and Methods

### Sample Collection

*Stenotrophomonas maltophilia* isolates were obtained from microbiology laboratories in different hospitals, in Alexandria, Egypt during a period of 8 months. Identification of the collected isolates was initially done using conventional biochemical methods, then confirmed by Vitek-2 (bioMérieux, France). Susceptibility testing for the isolates was carried out using disc diffusion method according to the CLSI guidelines [10], the antibiotics used were sulfamethoxazole/trimethoprim, minocycline and levofloxacin.

### Investigation of Virulence Genes

The bacterial DNA was extracted by boiling method, as described previously [11]. PCR was used to detect different virulence-associated genes. These include genes which encode protease enzymes (*stmPr-1*, *stmPr-2*), esterase enzyme (*smlt3773 locus*) and genes-associated with biofilm formation (*smf-1*, *spgM*, *rmlA* and *rpfF*). The different primers used in this study are detailed in Table (T-1) in supplementary document (SD1), together with the corresponding amplicon sizes, annealing temperatures and target genes. All primers were purchased from Invitrogen (Thermo Fisher Scientific, California, USA). The PCR master mix used was DreamTaq Green PCR Master Mix (Thermo Fisher Scientific, California, USA). PCR was performed on Biometra T-personal Thermal cycler (Analytik Jena GmbH, Jena, Germany). The amplification was done as follows: activation at 95 °C for 3 min, then 40 cycles of denaturation, annealing and extension, followed by a final extension step at 72 °C for 7 min. The 40 cycles included: denaturation at 95 °C for 30 s, annealing for 30 s, at the temperatures demonstrated in supplementary document (SD1), and extension at 72 °C for 1 min per 1000 bp. For the detection of each gene, we used 12.5 μl of the master mix, 1 μl of each primer (the concentration of each primer was 10 pmol), 2 μl of the previously extracted bacterial DNA, and sterile nuclease-free water was added so that the total volume of the reaction becomes 25 μl. Then, the amplicons were separated using gel electrophoresis on (2%) agarose gel, which contained 0.5 μg/mL ethidium bromide, and the bands were visualized using UV transilluminator (Bio-Rad, California, USA).

### Determination of the Antimicrobial Activity of Ascorbic Acid Against Planktonic Culture

The minimum inhibitory concentrations (MICs) of ascorbic acid, against the *S. maltophilia* clinical isolates and *S. maltophilia* ATCC 13,637 (Oxoid, London, UK), were determined using broth microdilution method [12]. The range of concentrations of ascorbic acid used was 100 to 0.09765 mg/ml. For each tested isolate, the inoculum was spectrophotometrically adjusted to 1.5 × 10^8^ CFU/ml (OD_600_ 0.12–0.13) and diluted to create a final concentration of 5 × 10^5^ CFU/ml in the microtiter plate [13, 14]. Then, the plates were incubated for 18–20 h at 37 °C. This was performed in triplicates [15].

### Determination of Biofilm-Forming Capacity and Antibiofilm Activity of Ascorbic Acid

Each well, of 96-well flat-bottomed microtiter plate, received 100 µl of ascorbic acid solution (1/8 MIC, 1/4 MIC and 1/2 MIC and MIC) and 100 µl of overnight broth subculture of the tested isolate diluted in sterile trypticase soy broth (TSB) (Oxoid, London, UK) corresponding to 1.5 × 10^8^ CFU/ml [13, 14]. This was performed in triplicates [15]. Positive controls containing no ascorbic acid and negative controls containing no bacteria were included [15]. The plates were incubated at 35 °C for 24 h [15]. Then, the medium was discarded and each well was washed three times with phosphate buffer saline (PBS) (pH 7.2) (Sigma-Aldrich, Milan, Italy) and biofilm samples were fixed by incubating the microtiter plates at 60 °C for 1 h [16, 17]. The wells were stained with crystal violet [17]. Then, the dye, which is bound to the biofilm was extracted with ethanol 99.5% [16]. The optical density (OD) of each well was measured at 590 nm and the isolates were categorized as non-biofilm producers, weak, moderate or strong biofilm producers based on the measured OD, as described before by Stepanovic et al [18]. Then, the percentage of the inhibition of the biofilm formation was calculated as described previously by Jadhav et al. [19].

### Scanning Electron Microscope (SEM)

One of the isolates (S5) was grown in TSB and TSB supplemented with MIC (3.125 mg/ml) of ascorbic acid in six-well polystyrene plate. The SEM examination and image capturing was carried out using JSM-IT200 (JEOL, Japan), after fixing the adherent cells in 2.5% glutaraldehyde in PBS (PH 7.2), gradual dehydration by ethanol and gold coating the samples as described before by Gad et al [20].

### Statistical Analysis

Statistical analysis of the data were performed using IBM SPSS software package version 20.0. (Armonk, NY: IBM Corp) Significance of the results was assessed at the 5% level. The used tests were Chi-square test and Fisher’s Exact. One way ANOVA test was done using Post Hoc Test (Tukey), and the significance of the results was, also, assessed at the 5% level.

## Results

A total of 20 *S. maltophilia* isolates, were collected from different clinical samples, including respiratory tract infections, blood stream infections and wound infections. The different type of samples corresponding to each isolate is shown Table (T-2) in supplementary document (SD2).

The susceptibility of the different strains to sulphamethoxazole/trimethoprim, levofloxacin and minocycline is shown in Table 1.

**Table 1:** Susceptibility pattern of the 20 *S. maltophilia* isolates

Antimicrobial	Resistant		Intermediate		Sensitive	
	No	%	No	%	No	%
Trimethoprim/Sulfamethoxazole	11	55	8	40	1	5
Minocycline	3	15	14	70	3	15
Levofloxacin	6	30	2	10	12	60

### Genotypic Investigation of Virulence Genes

Genotypic investigation showed that the genes responsible for proteases and esterase activity were present among the isolates. We found that the serine proteases genes *stmPr1* (1621 bp), *stmPr1*(868 bp) and *stmPr2* were present in 70%, 15% and 75%, respectively. On the other hand, esterase coding gene *smlt-3773 locus* was present in 95% of the isolates as shown in Table 2. Moreover, Genes associated with biofilm formation were present. *smf-1*, *rpfF*, *rmlA* and *spgM*, were found in (90%), (45%), (85%) and (30%) of the isolates, respectively, as demonstrated in Table 2. The figures of the different bands are shown in supplementary document (SD3).

**Table 2:** Genes encoding virulence enzymes and biofilm formation

	Genes encoding proteolytic enzymes			Gene encoding esterase	Genes associated with biofilm formation			
	*stmPr1* (1621 bp)	*stmPr1 *(868 bp)	*stmPr2*	*smlt-3773 locus*	*Smf-1*	*rmlA*	*spgM*	*rpfF*
Positive	14 (70%)	3* (15%)	15 (75%)	19 (95%)	18 (90%)	17 (85%)	6 (30%)	9 (45%)
Negative	6 (30%)	17 (85%)	5 (25%)	1 (5%)	2 (10%)	3 (15%)	14 (70%)	11 (55%)

*The 3 isolates were found to harbor *stmPr−1* using both primers

### Correlation Between Biofilm-Forming Ability and the Presence of Genes Associated with BIOFILM Formation

The extent of biofilm formation among *S. maltophilia* isolates was very high. The majority of the isolates were strong biofilm producers 15 (75%) and 5 (25%) were moderate biofilm producers. None of the isolates were either weak biofilm producer nor non- biofilm producer. The biofilm producing ability of each isolate is demonstrated in Table 3.

**Table 3:** Characterization of the different isolates according to their biofilm production and the presence of the genes associated with biofilm formation

Isolate	Biofilm formation	Genes associated with biofilm formation				Esterase coding gene	Genes encoding proteolytic enzymes		
		*Smf-1*	*rmlA*	*spgM*	*rpfF*	*smlt-3773 locus*	*stmPr1* (1621 bp)	*stmPr1* (868 bp)	*stmPr2*
S1	Strong	Positive	Positive	Positive	Positive	Positive	–	–	Positive
S2	Strong	Positive	Positive	Positive	Positive	Positive	Positive	–	Positive
S3	Strong	Positive	–	–	–	Positive	–	–	Positive
S4	Strong	Positive	–	–	–	Positive	Positive	Positive	–
S5	Strong	Positive	Positive	–	–	Positive	Positive	Positive	Positive
S6	Strong	Positive	Positive	–	–	Positive	Positive	–	–
S7	Strong	Positive	Positive	–	Positive	Positive	Positive	–	Positive
S8	Moderate	–	Positive	Positive	Positive	Positive	Positive	Positive	Positive
S9	Moderate	Positive	Positive	–	–	Positive	–	–	–
S10	Moderate	Positive	Positive	–	–	Positive	–	–	Positive
S11	Strong	Positive	Positive	–	Positive	–	Positive	–	–
S12	Strong	Positive	Positive	–	Positive	Positive	–	–	Positive
S13	Strong	Positive	Positive	Positive	Positive	Positive	Positive	–	Positive
S14	Strong	Positive	Positive	–	–	Positive	Positive	–	Positive
S15	Strong	Positive	Positive	Positive	Positive	Positive	Positive	–	Positive
S16	Moderate	Positive	Positive	Positive	Positive	Positive	Positive	–	Positive
S17	Strong	Positive	–	–	–	Positive	–	–	Positive
S18	Moderate	Positive	Positive	–	–	Positive	Positive	–	Positive
S19	Strong	Positive	Positive	–	–	Positive	Positive	–	Positive
S20	Strong	–	Positive	–	–	Positive	Positive	–	–

Statistically, there was no significant correlation between the presence of *smf-1*, *rpfF*, *rmlA* or *spgM* and the extent of biofilm formation, and this is shown in Table 4.

**Table 4:** Correlation between biofilm formation and the biofilm-associated genes

Genes associated	Biofilm formation				*P* value
	Moderate (*n* = 5)		Strong (*n* = 15)		
	No	%	No	%	
*Smf-1*	4	80.0	14	93.3	0.447^*^
*rmlA*	5	100.0	12	80.0	0.539^*^
*spgM*	2	40.0	4	26.7	0.613^*^
*rpfF*	2	40.0	7	46.7	1.000^*^

*p*: *p* value (for Chi−square test), to compare between the two groups

*Statistically insignificant (*p* value > 0.05)

### The Effect of Ascorbic Acid on Planktonic Culture and its Biofilm Inhibition Activity

The MIC values of ascorbic acid against the *S. maltophilia* clinical isolates and *S. maltophilia* ATCC 13,637 ranged from 0.78 to 50 mg/ml. The modal MIC was 3.125 mg/ml. The MIC_50_ and MIC_90_ was 3.125 mg/ml and 6.25 mg/ml, respectively, the MIC value for each isolate is shown in Table 5. The MIC value of ascorbic acid against *S. maltophilia* ATCC 13,637 was 1.5625 mg/ml, as shown in Table 5.

**Table 5:** Comparison between ascorbic acid MIC and the biofilm inhibition effect of different concentrations of ascorbic acid

Isolate	MIC in mg/ml	Percentage of biofilm inhibition of ascorbic acid at different concentrations in mg/ml (MIC, 1/2 MIC, 1/4 MIC and 1/8 MIC)			
		MIC (%)	1/2 MIC (%)	1/4 MIC	1/8 MIC
S1	1.5625	82	80.83	48%	53%
S2	1.5625	92.7	81.2	60.3%	48%
S3	3.125	71	62.1	50%	21%
S4	1.5625	58.7	56	56%	41.4%
S5	3.125	86	76	41%	9.5%
S6	3.125	84.4	75.5	37.8%	37.7%
S7	0.09	38	3.5	ND*	ND*
S8	1.5625	77.4	71.7	70%	53.3%
S9	3.125	84.6	61.6	53.8%	38.5%
S10	3.125	87.5	62.4	53.8%	11.2%
S11	3.125	59.1	37.8	19.7%	6.6%
S12	3.125	64.1	48.5	43.2%	17.6%
S13	3.125	51	54.7	34.7%	5.7%
S14	25	70.6	61.7	52.9%	49.2%
S15	6.25	61	36.4	16.4%	5.8%
S16	0.78	89	81.7	71.2%	35%
S17	6.25	78.1	69.3	61.6%	63.8%
S18	1.5625	85.5	67.8	45.2%	11.3%
S19	3.125	68.2	67.5	76.1%	68.2%
S20	50	48.5	33.94	26.9%	12.2%
ATCC 13,637	1.5625	95	90	70%	30%

*ND means “not detected”

**Fig. 1:**
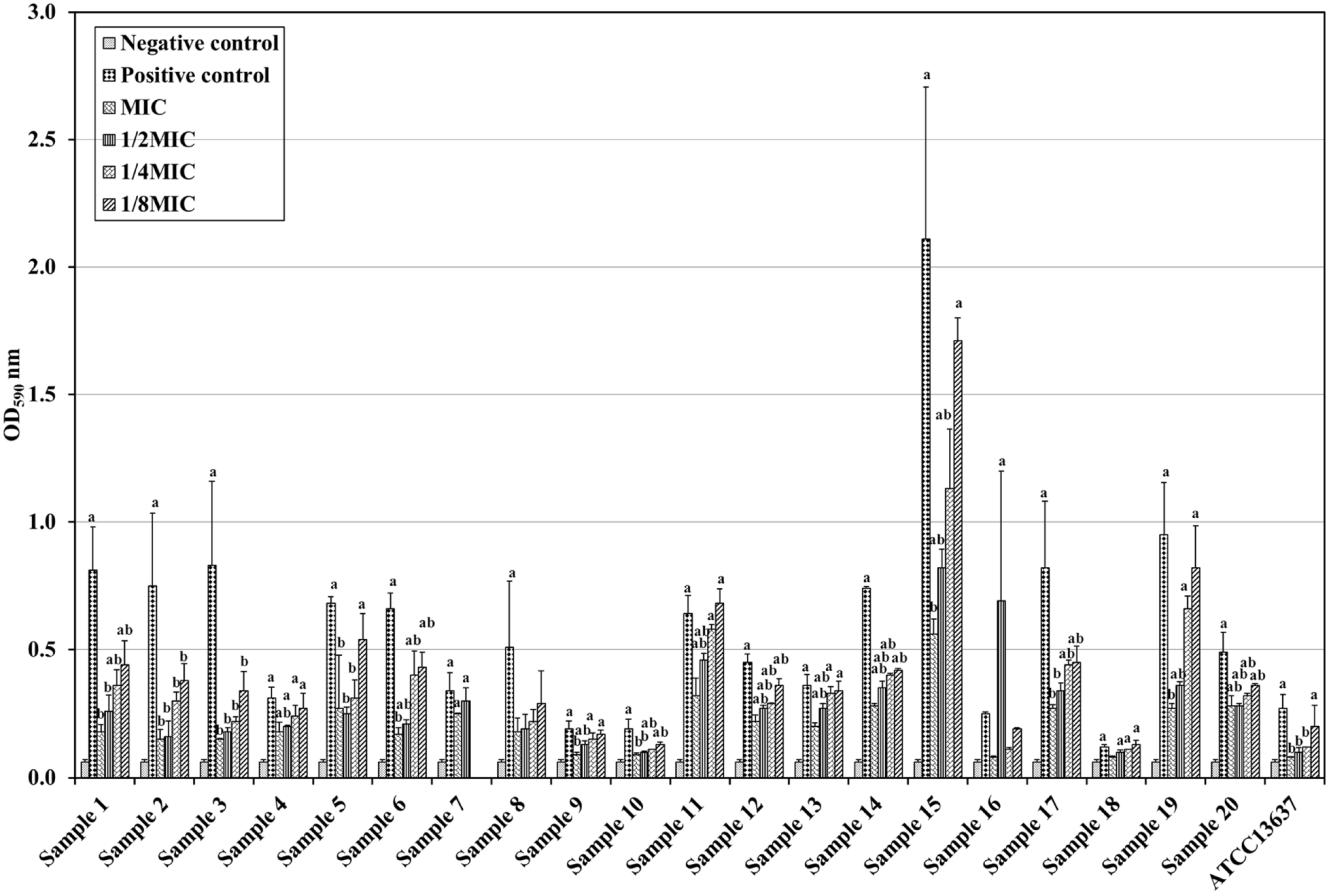
The effect of ascorbic acid on the biofilm-forming capacity of *S. maltophilia*. Three replica for each group; Data were expressed using Mean ± SD. (SE); *SD* standard deviation, *SE* standard error of mean, *F* F for one way ANOVA test, Pairwise comparison bet. each 2 groups was done using Post Hoc Test (Tukey); *p* *p* value for comparing between the studied groups; *: Statistically significant at *p* ≤ 0.05; **a** significant with negative control; **b** significant with positive control; the positive control was the isolate cultured in TSB without ascorbic acid and the negative control was the media without the cultured bacteria

**Fig. 2:**
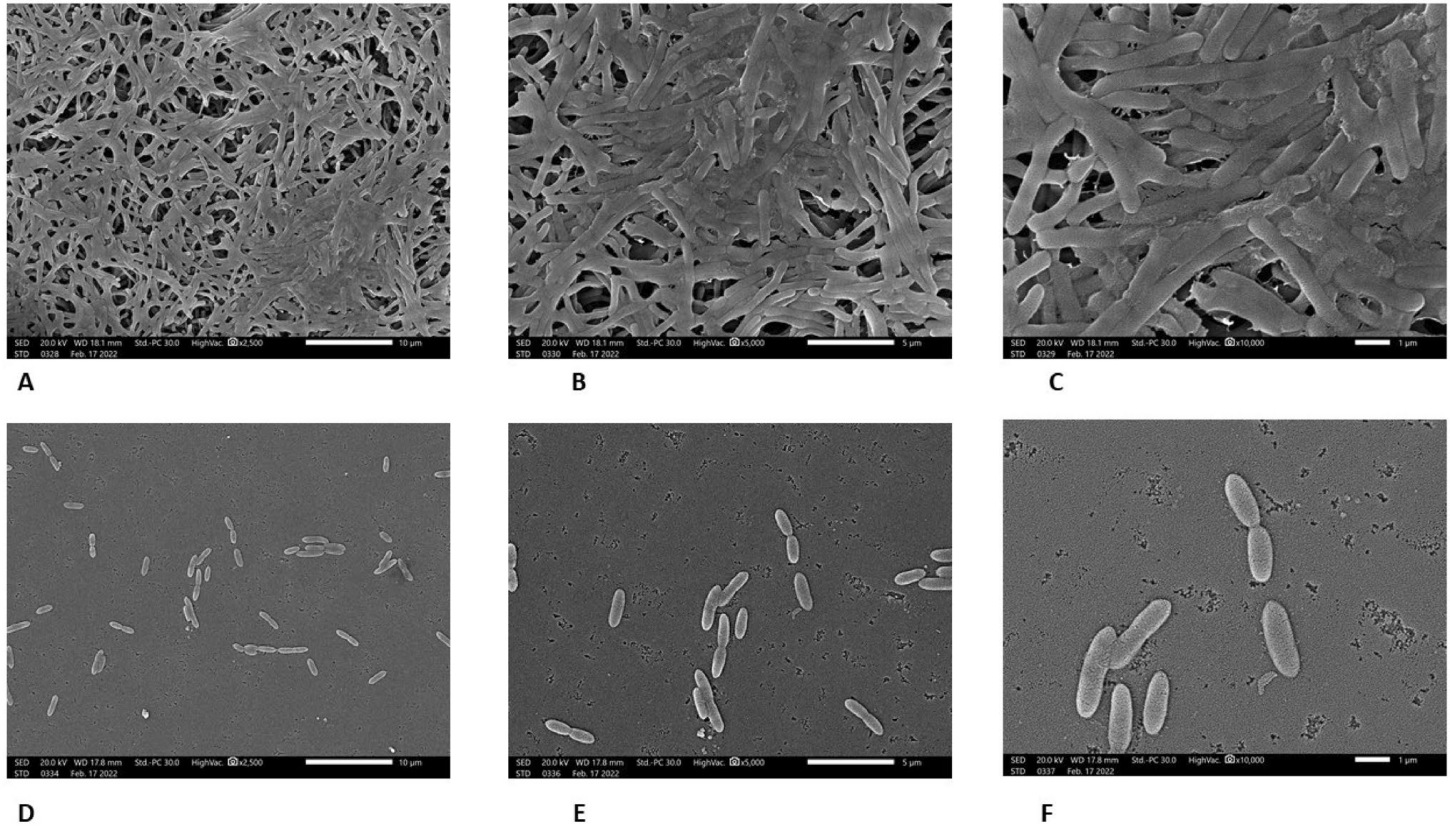
SEM images showing **A**, **B** and **C** which demonstrate isolate (S5) in TSB with different magnifications X2,500, X5000, X10,000, respectively. SEM images showing **D**, **E** and **F**, which demonstrate isolate (S5) in MIC (3.125 mg/ml) of ascorbic acid in TSB with different magnifications X2,500, X5000, X10,000, respectively

To determine the inhibitory effect of ascorbic acid on biofilm formation, the biofilm-forming ability of *S. maltophilia* was determined in the presence of variable concentrations of ascorbic acid as well as in the absence of ascorbic acid. The inhibition of biofilm formation was found to be concentration dependent. Similar concentration-dependent manner was shown by *S. maltophilia* ATCC 13,637, as shown in Fig. 1 and the Table (T-3) in supplementary document SD4. The highest percentage of biofilm inhibition was more evident with MIC, this was shown by the SEM in Fig. 2.

## Discussion

*Stenotrophomonas maltophilia* has acquired a lot of interest due to the growing number of nosocomial infections caused by this emerging pathogen [2]. Biofilm formation is considered the corner stone for establishing infections in many bacteria including *S. maltophilia* [7].

The aim of this study was the genotypic characterization of the different virulence-associated genes and the investigation of the effect of ascorbic acid on *S. maltophilia* biofilm formation.

Many of the isolates harbored both serine proteases genes; 70% of the isolates harbored *stmPr1* (K279a allelic variant of SmtPr1), and 75% harbored *stmPr-2*. Major (StmPr1) and minor (StmPr2) serine proteases are secreted by Xps, a T2SS in *S. maltophilia* [3]. Duan et al., showed that s*tmPr1* and s*tmPr2* were present in (79.6%) and (95.4%), respectively [21].

Most of the isolates possessed *smlt-3773 locus* which codes for an esterase enzyme. Nicoletti et al., demonstrated that among all their isolates only three environmental isolates did not harbor *smlt3773 locus* [4]. Duan et al., reported that *smlt3773 locus* was present in (52.7%) of their isolates, while in another chinese study, it was found in (91.3%) of the *S. maltophilia* from pediatric patients [21, 22].

Most of the isolates harbored *smf-1*, which encodes for type-1 fimbriae, which plays a significant role in adherence to surfaces and the early stages of biofilm formation [2, 23]. Nicoletti et al., reported that *smf-1* was present in all their clinically derived *S. maltophilia* isolates [4]. Azimi et al., showed that *smf-1* was present in (99.3%) of their isolates [13].

*rpfF* plays a critical role in the production of the diffusable signal factor (DSF), which mediates quorum sensing in *S. maltophilia*. Moreover, disruption to *rpfF* hampers DSF synthesis and inhibits levels of extracellular proteases [16, 24]. On the other hand, *spgM* encodes an enzyme with both phosphoglucomutase (PGM) activity as well as a phosphomannomutase activity. *spgM* has been implemented in biofilm formation [6]. *rmlA* is aslo associated with attachement and biofilm formation [6]. Among the 20 isolates, *rpfF*, *rmlA* and *spgM* were also investigated, they were found in (45%), (85%) and (30%) of the isolates, respectively. Azimi et al., demonstrated that *rpfF*, *rmlA* and *spgM* were present in (70%), (98%), and (97.3%), respectively [13]. Bostanghadiri et al., reported that *rpfF*, *rmlA* and *spgM* were present in (89.41%), (84.71%) and (100%), respectively [25]. Another study, reported that *rpfF*, *rmlA* and *spgM* were present in (45.2%), (83.7%) and (100%), respectively [22].

The ability to form biofilm was predominant among the 20 *S. maltophilia* isolates; all the isolates were biofilm producers; 75% were strong biofilm producers and 25% were moderate biofilm producers. Several reports have also witnessed an increased incidence of biofilm-forming ability among their isolates. Azimi et al*.* and Bostanghadiri et al*.* reported that (98.7%) and (95.7%) of their isolates, respectively, were biofilm producers with variable capacity of biofilm formation [5, 13]. Zhou et al*.* showed predominance of biofilm formation (100%) among their isolates, where only 2 isolates were classified as weak biofilm producers [6]. A study based on five European countries by Pompolio et al*.* revealed that (91.7%) of the isolates tested were biofilm producers categorized into different groups according to the extent of biofilm formation [17].

We found that there was no significant correlation between the presence of *smf-1*, *rpfF*, *rmlA* or *spgM* and the extent of biofilm formation.

Biofilms allow bacterial cells, to adhere and persist on both biotic and abiotic surfaces, and consequently to establish infections in both community and hospital settings [2]. Different studies have tackled hindering biofilm formation, however many of them used antibacterial agents which may further complicate the antimicrobial resistance challenge [26, 27]. In this context, we decided to evaluate the ability of ascorbic acid to inhibit biofilm formation among the 20 *S. maltophilia* clinical isolates as well as *S. maltophilia* ATCC 13,637. Ascorbic acid, also known as vitamin C, is antioxidant and a micronutrient, which is needed to sustain general health and immune system functions [9].

The MIC values of ascorbic acid ranged from 0.78 to 50 mg/ml. The MIC_50_ and MIC_90_ was 3.125 mg/ml and 6.25 mg/ml respectively. Kwiecińska-Piróg et al. and Verghese et al*.* showed that a concentration of 10 mg/ml of ascorbic acid can efficiently inhibit growth among Enterobacteriaceae [28, 29]. Another study conducted by Mumtaz et al., demonstrated that Vitamin C notably hindered the growth of some Gram-positive as well as some Gram-negative bacteria including members of the Enterobacteriaceae family as well as *P. aeruginosa* [30]. Other reports also investigated the inhibitory effect of vitamin C on several other bacteria including *M. tuberculosis* and *H. pylori*, none of which tested the effect of vitamin C on *S. maltophilia* [31–33]. To the best of our knowledge, this is the first study to evaluate the effect of ascorbic acid on *S. maltophilia* biofilm formation. Ascorbic did not induce biofilm formation, at sublethal doses, in fact it inhibited biofilm formation in *S. maltophilia* in a concentration-dependent manner. Interestingly, the highest percentage of biofilm inhibition was more evident with MIC. This is consistent with previous reports by Mumtaz et al. [30] and Eydou et al. [9].

## Conclusion

Biofilm formation in *S. maltophilia* represents a therapeutic and a clinical challenge. Biofilm inhibition remains a reasonable solution to meet this challenge. Here, we conclude that Vitamin C can inhibit biofilm formation in *S. maltophilia* in a concentration-dependent manner. Selecting biofilm inhibitors such as vitamin C and other non-chemotherapeutic agents, spares the use of common antibacterial agents as biofilm inhibitors which paves the way for better clinical outcomes.

## Supplementary Information

Below is the link to the electronic supplementary material.

Supplementary file1 (DOCX 20 kb)

Supplementary file2 (DOCX 14 kb)

Supplementary file3 (PPTX 2764 kb)

Supplementary file4 (DOCX 17 kb)

## Data Availability

Not applicable.
