# Supplementary material for: Stenotrophomonas maltophilia: Genotypic Characterization of Virulence Genes and The Effect of Ascorbic Acid on Biofilm Formation
Source: Curr Microbiol. 2022 May 5;79(6):180. doi: 10.1007/s00284-022-02869-7 (PMC9068641; doi:10.1007/s00284-022-02869-7)
Supplement: Supplementary file 1 — Supplementary file1 (DOCX 20 kb) [file 284_2022_2869_MOESM1_ESM.docx]

**Table (T-1): The different primers used in this study.**

| **Primer** | **Nucleotide Sequence**  **(5'–3')** | **Target** | **Annealing temperature in °C** | **Amplicon Size (bp)** | **Reference** |
| --- | --- | --- | --- | --- | --- |
| *smf-1* For | GGAAGGTATGTCCGAGTCCG | *smf-1* | 54 | 674 | [4] |
| *smf-1* Rev | GCGGGTACGGCTACGATCAGTT |  |  |  |  |
| *stmPr-1a* For | GCC GCA GTG TTG GTT CGA TCC A | *stmPr1*  (*stmPr1* allelic variant of strain K279a) | 60 | 1621 | [4] |
| *stmPr-1a* Rev | CAG TTC TCG GTG CAC GGC TCT T |  |  |  |  |
| *stmPr-1* For | CACGGCGGTCTT GTTGGTCA | *stmPr1*  (Internal fragment of the *stmPr1* gene) | 58 | 868 | [4] |
| *stmPr-1* Rev | CGAGAACGACAACGAGTGCTACA |  |  |  |  |
| *stmPr2* For | GCCGATTCCGGCATTCACACC | *stmPr2*  gene of strain K279a | 59 | 1764 | [4] |
| *stmPr2* Rev | GGTCAGGCCCGAGAAGGTGCT |  |  |  |  |
| *Smlt3773* For | CGGTGCCGAACTCGTAACCGG | *smlt3773 locus* | 54 | 1342 | [4] |
| *Smlt3773* Rev | CTTCCGGCCATGGCAGGCGAA |  |  |  |  |
| RPFF For | GCAGAAGACCAACGTCGGCAAG | *rpfF* | 57 | 700 | [24] |
| RPFF Rev | CTTCCTAGGCGACGATGGTGTG |  |  |  |  |
| *rmlA* For | CGGAAAAGCAGAACATCG | *rmlA* | 49 | 1222 | [6] |
| *rmlA* Rev | GCAACTTGGTTTCAATCACTT |  |  |  |  |
| *spgM* For | ATACCGGGGTGCGTTGAC | *spgM* | 53 | 2750 | [6] |
| *spgM* Rev | CATCTGCATGTGGATCTCGT |  |  |  |  |
