# Supplementary material for: Stenotrophomonas maltophilia: Genotypic Characterization of Virulence Genes and The Effect of Ascorbic Acid on Biofilm Formation
Source: Curr Microbiol. 2022 May 5;79(6):180. doi: 10.1007/s00284-022-02869-7 (PMC9068641; doi:10.1007/s00284-022-02869-7)
Supplement: Supplementary file 2 — Supplementary file2 (DOCX 14 kb) [file 284_2022_2869_MOESM2_ESM.docx]

**Table (T-2): Additional information about the source of isolates and the type of samples from which the isolates were obtained.**

| **Isolate** | **Source** | **Hospital** |
| --- | --- | --- |
| **S1** | **Blood** | **H1** |
| **S2** | **Blood** | **H1** |
| **S3** | **Wound Swab** | **H1** |
| **S4** | **Sputum** | **H1** |
| **S5** | **Blood** | **H1** |
| **S6** | **Blood** | **H1** |
| **S7** | **Wound Swab** | **H1** |
| **S8** | **Sputum** | **H1** |
| **S9** | **Sputum** | **H1** |
| **S10** | **MiniBAL** | **H1** |
| **S11** | **MiniBAL** | **H1** |
| **S12** | **Sputum** | **H1** |
| **S13** | **Blood** | **H2** |
| **S14** | **Wound Swab** | **H2** |
| **S15** | **Blood** | **H3** |
| **S16** | **Sputum** | **H3** |
| **S17** | **Blood** | **H3** |
| **S18** | **Wound Swab** | **H3** |
| **S19** | **MiniBAL** | **H3** |
| **S20** | **Blood** | **H3** |

**H1: Mabaret El-Asafra Hospital; H2: Medical Research Institute Hospital; H3: Alexandria Main University Hospital.s**
