# Supplementary material for: Stenotrophomonas maltophilia: Genotypic Characterization of Virulence Genes and The Effect of Ascorbic Acid on Biofilm Formation
Source: Curr Microbiol. 2022 May 5;79(6):180. doi: 10.1007/s00284-022-02869-7 (PMC9068641; doi:10.1007/s00284-022-02869-7)
Supplement: Supplementary file 3 — Supplementary file3 (PPTX 2764 kb) [file 284_2022_2869_MOESM3_ESM.pptx]

## Slide 1
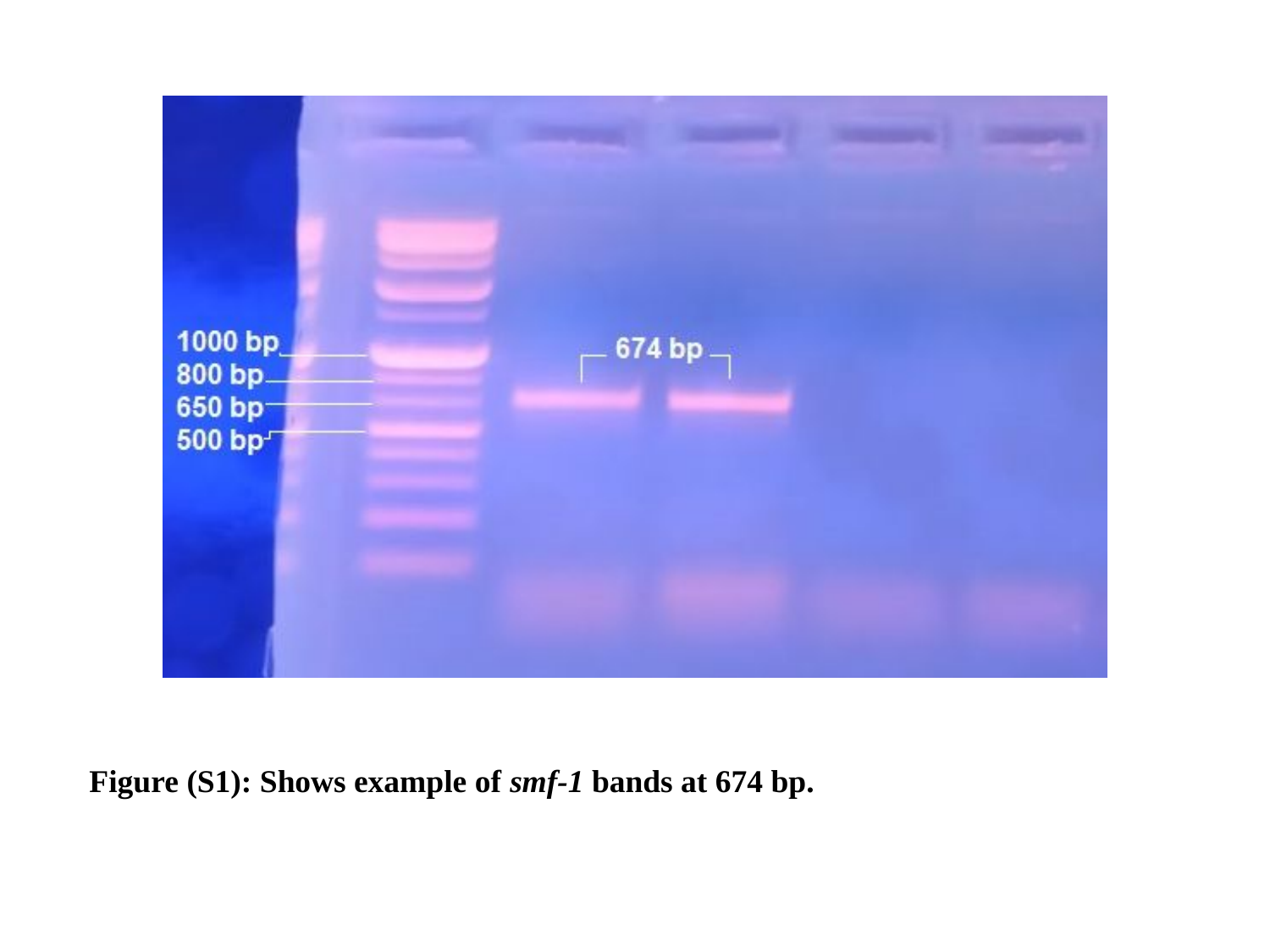

Figure (S1): Shows example of smf-1 bands at 674 bp.

## Slide 2
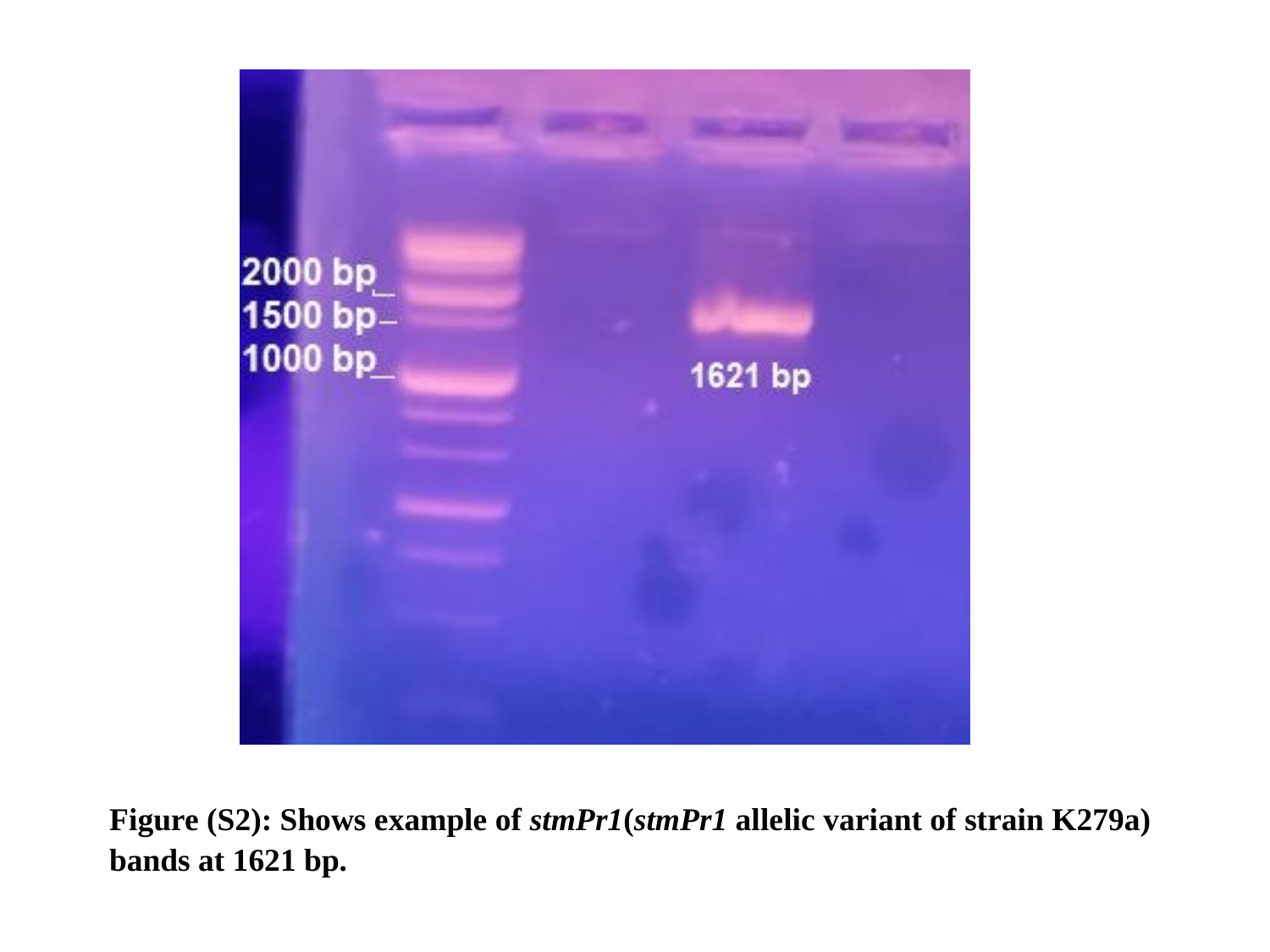

Figure (S2): Shows example of stmPr1(stmPr1 allelic variant of strain K279a) bands at 1621 bp.

## Slide 3
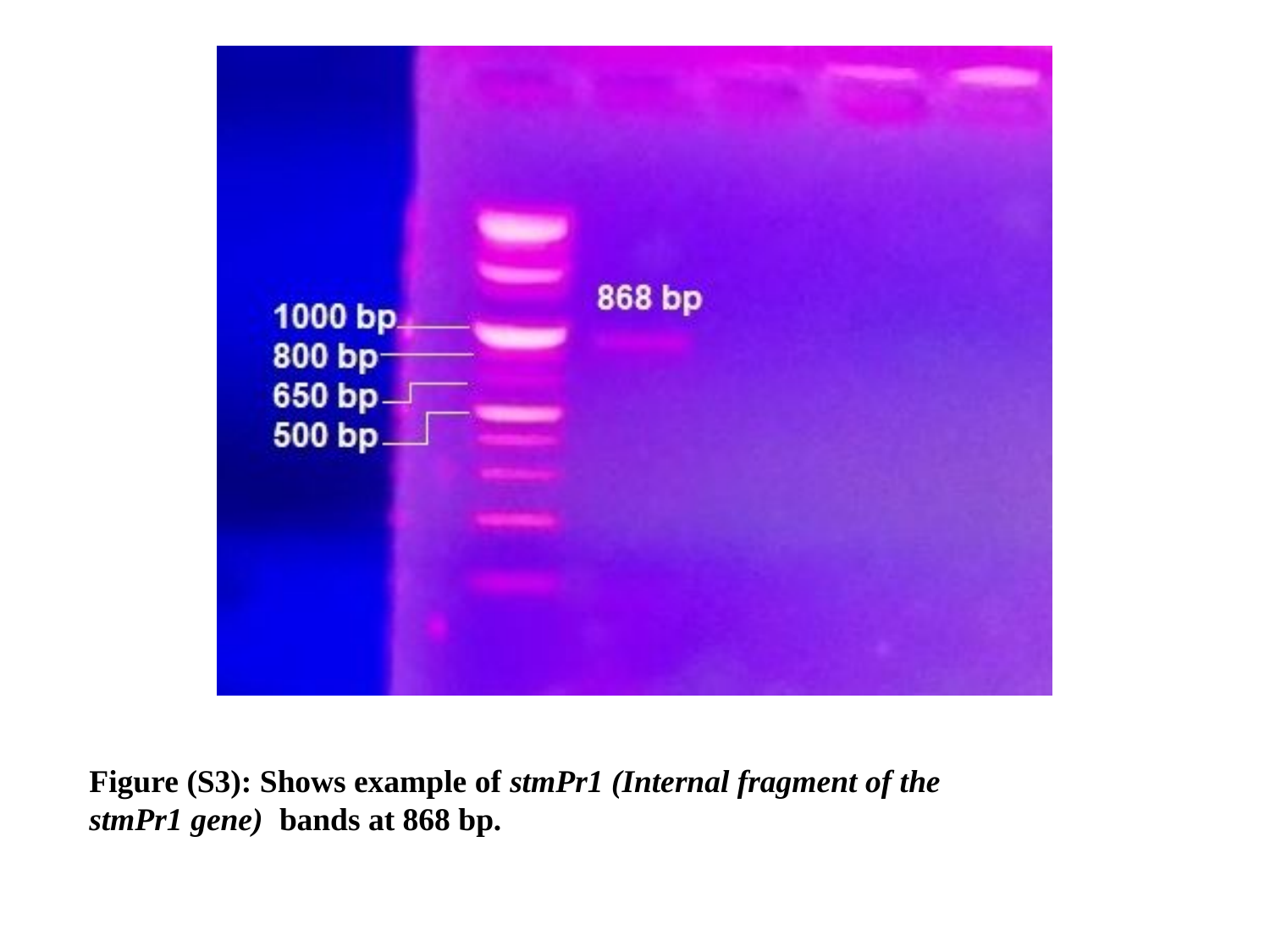

Figure (S3): Shows example of stmPr1 (Internal fragment of the stmPr1 gene) bands at 868 bp.

## Slide 4
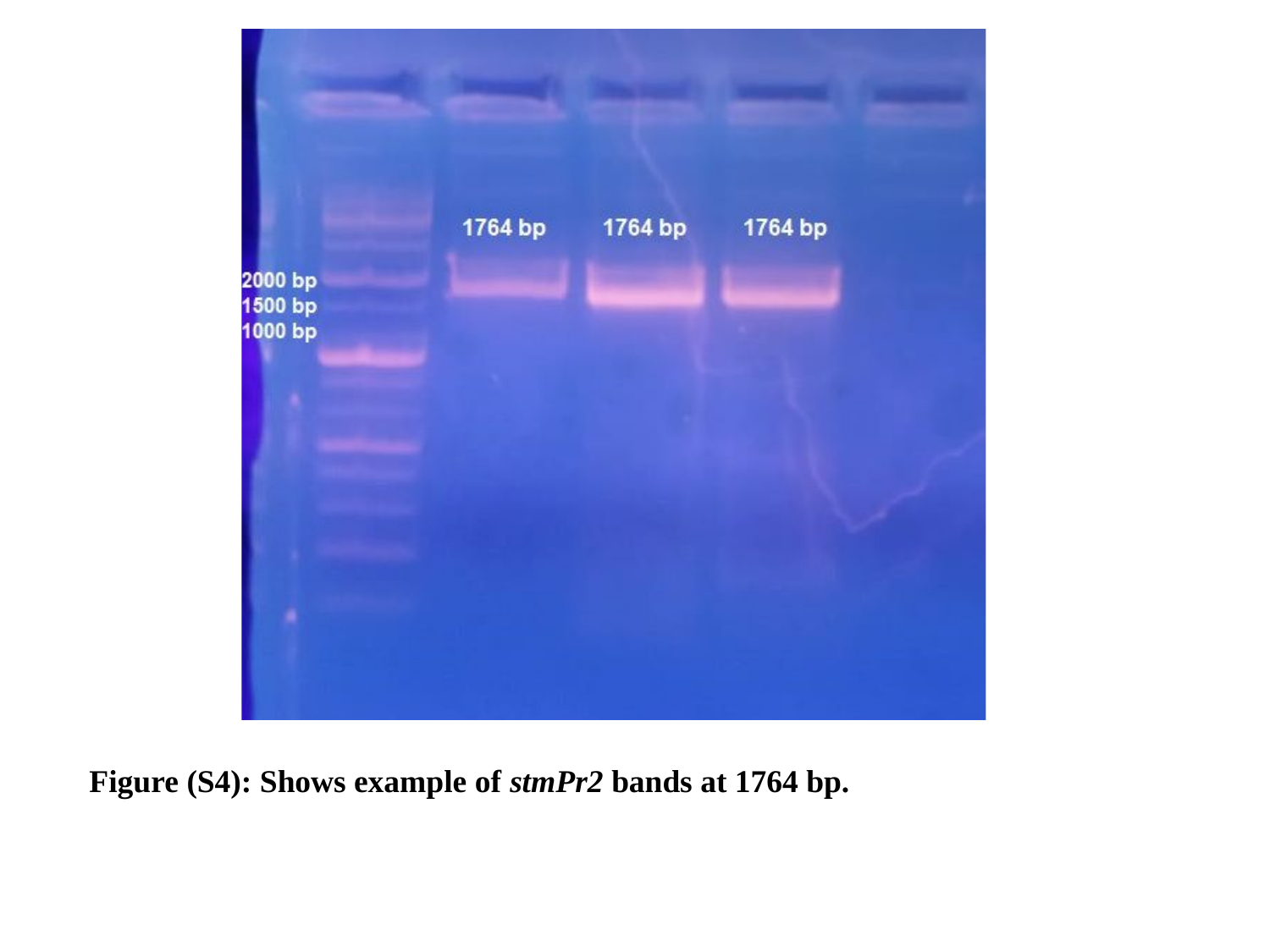

Figure (S4): Shows example of stmPr2 bands at 1764 bp.

## Slide 5
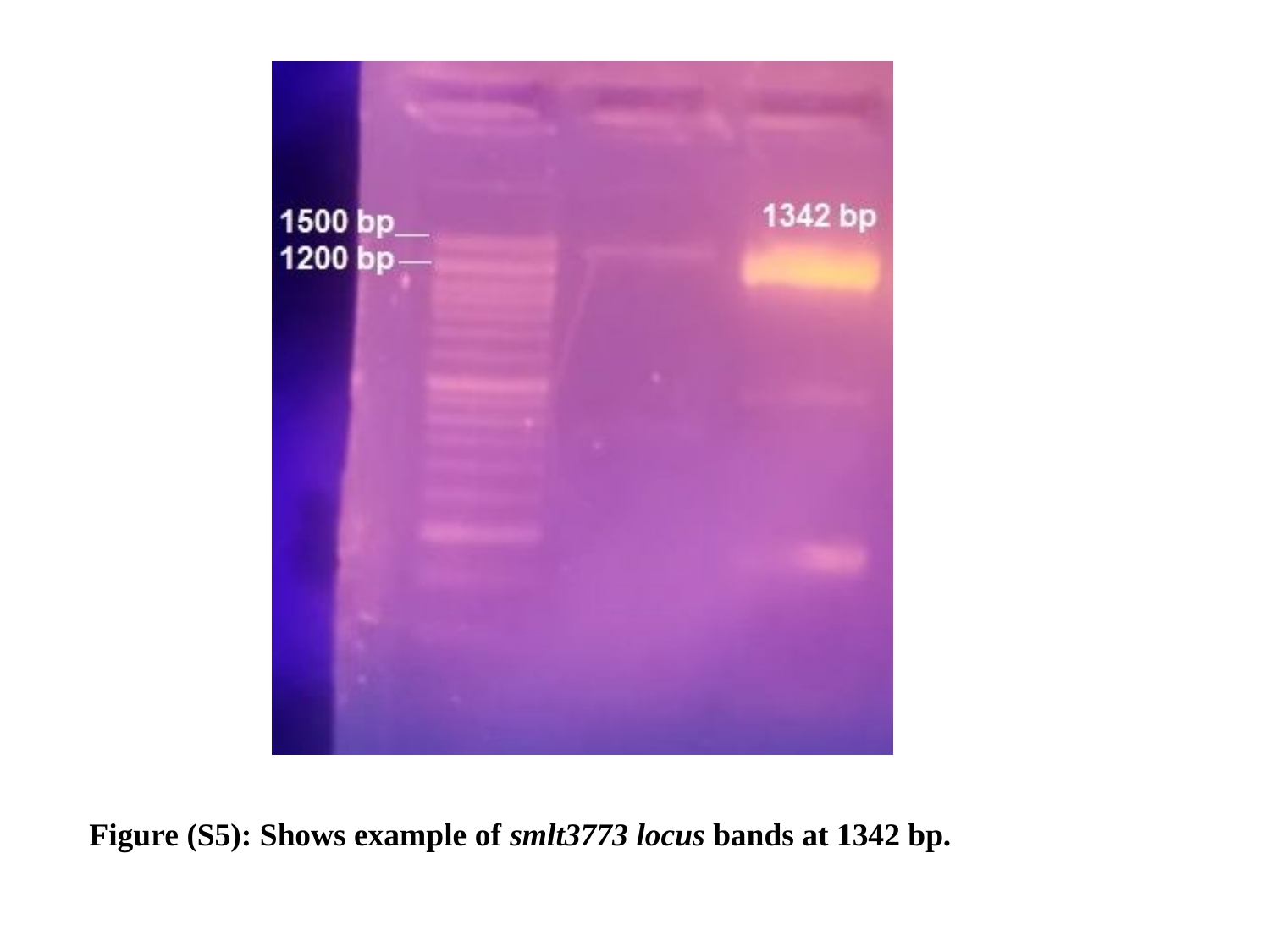

Figure (S5): Shows example of smlt3773 locus bands at 1342 bp.

## Slide 6
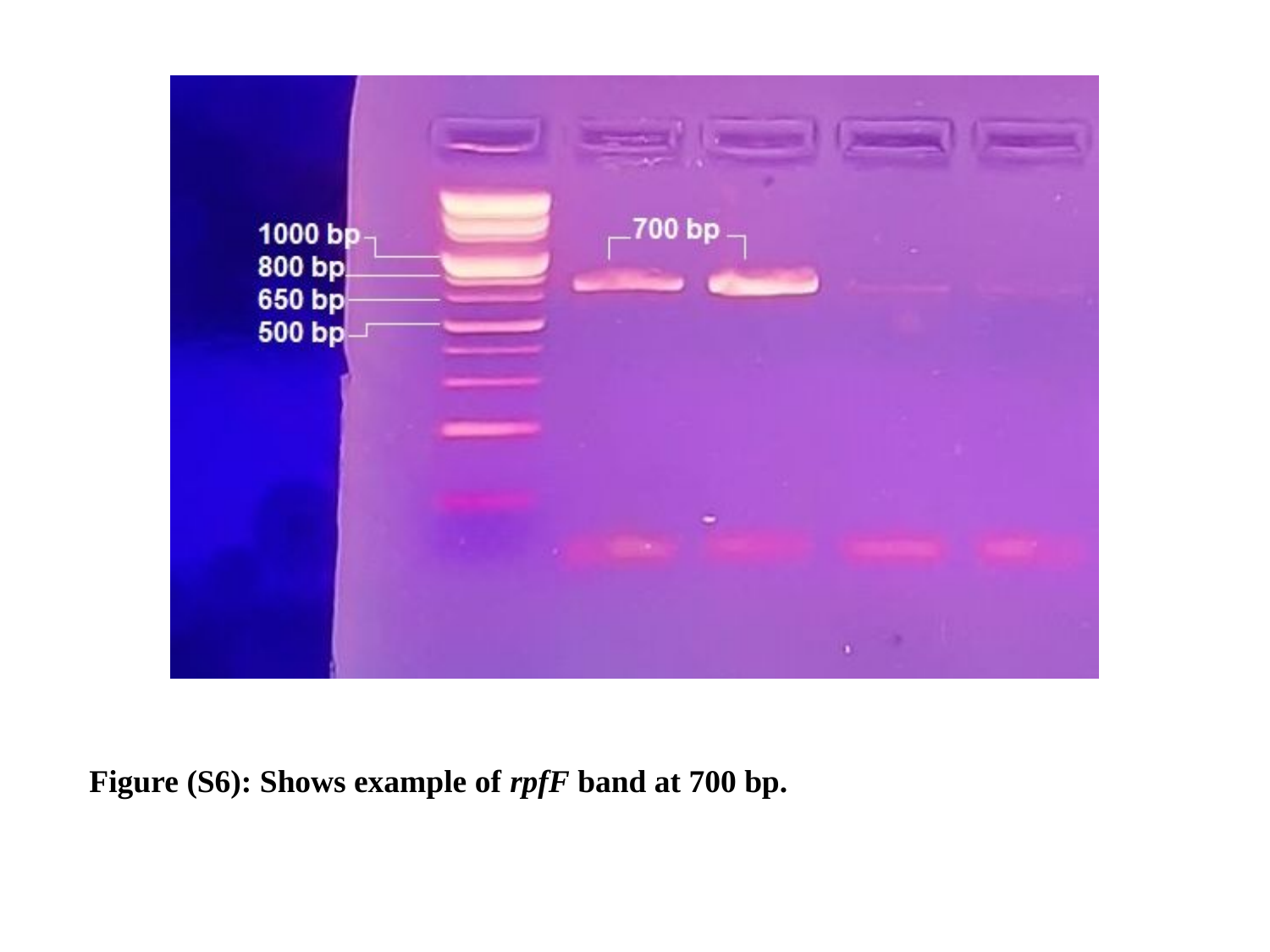

Figure (S6): Shows example of rpfF band at 700 bp.

## Slide 7
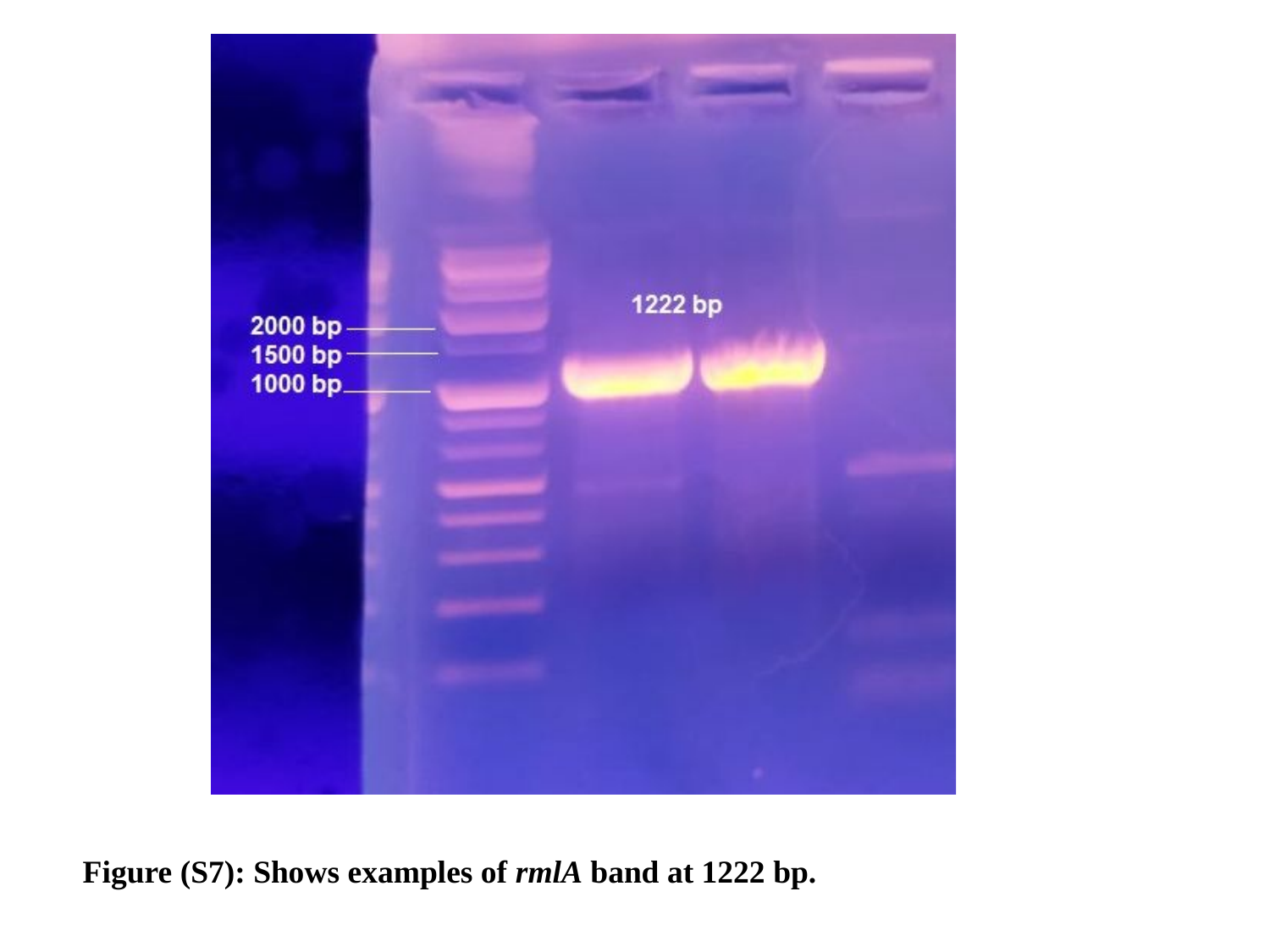

Figure (S7): Shows examples of rmlA band at 1222 bp.

## Slide 8
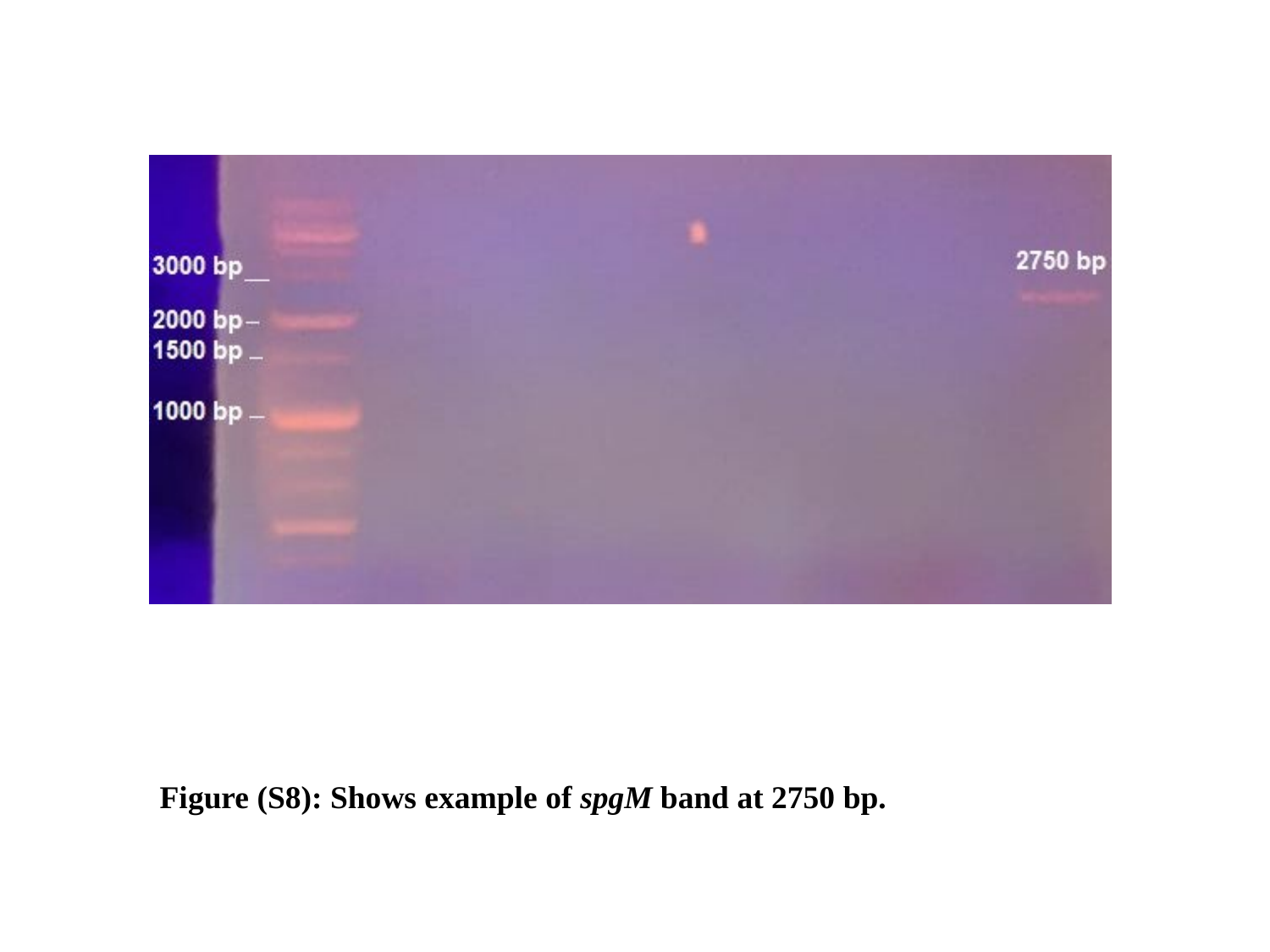

Figure (S8): Shows example of spgM band at 2750 bp.
