# Supplementary material for: Stenotrophomonas maltophilia: Genotypic Characterization of Virulence Genes and The Effect of Ascorbic Acid on Biofilm Formation
Source: Curr Microbiol. 2022 May 5;79(6):180. doi: 10.1007/s00284-022-02869-7 (PMC9068641; doi:10.1007/s00284-022-02869-7)
Supplement: Supplementary file 4 — Supplementary file4 (DOCX 17 kb) [file 284_2022_2869_MOESM4_ESM.docx]

**Table (T-3): The effect of ascorbic acid on the biofilm forming capacity of *S. maltophilia*.**

**Table (1): Comparison between the different studied groups according to OD_590_**

| **OD_590_ nm** | **Negative control** | **Positive control** | **MIC** | **1/2MIC** | **1/4MIC** | **1/8MIC** | **F** | **p** |
| --- | --- | --- | --- | --- | --- | --- | --- | --- |
| **Sample 1** | 0.06 ± 0.01 (0.006) | 0.81^a^ ± 0.170 (0.098) | 0.18^b^ ± 0.028 (0.016) | 0.26^b^ ± 0.064 (0.037) | 0.36^ab^±0.063 (0.036) | 0.44^ab^± 0.096 (0.055) | 25.891^*^ | <0.001^*^ |
| **Sample 2** | 0.06 ± 0.01 (0.006) | 0.75^a^ ± 0.284 (0.164) | 0.15^b^ ± 0.039 (0.022) | 0.16^b^ ± 0.062 (0.036) | 0.30^b^ ± 0.034 (0.020) | 0.38^b^ ± 0.065 (0.038) | 12.240^*^ | <0.001^*^ |
| **Sample 3** | 0.06 ± 0.01 (0.006) | 0.83^a^ ± 0.330 (0.190) | 0.15^b^ ± 0.003 (0.002) | 0.18^b^ ± 0.015 (0.008) | 0.22^b^ ± 0.018 (0.010) | 0.34^b^ ± 0.075 (0.043) | 12.176^*^ | <0.001^*^ |
| **Sample 4** | 0.06 ± 0.01 (0.006) | 0.31^a^ ± 0.044 (0.025) | 0.18^ab^ ±0.038 (0.022) | 0.20^a^ ± 0.005 (0.003) | 0.24^a^ ± 0.044 (0.025) | 0.27^a^ ± 0.061 (0.035) | 14.634^*^ | <0.001^*^ |
| **Sample 5** | 0.06 ± 0.01 (0.006) | 0.68^a^ ± 0.027 (0.016) | 0.27^b^ ± 0.209 (0.121) | 0.25^b^ ± 0.025 (0.015) | 0.31^b^ ± 0.071 (0.041) | 0.54^a^ ± 0.101 (0.059) | 14.576^*^ | <0.001^*^ |
| **Sample 6** | 0.06 ± 0.01 (0.006) | 0.66^a^ ± 0.061 (0.035) | 0.17^b^ ± 0.027 (0.016) | 0.21^ab^ ±0.017 (0.010) | 0.40^ab^ ±0.095 (0.055) | 0.43^ab^ ±0.060 (0.035) | 49.056^*^ | <0.001^*^ |
| **Sample 7** | 0.06 ± 0.01 (0.006) | 0.34^a^ ± 0.070 (0.040) | 0.25^a^ ± 0.003 (0.002) | 0.30^a^ ± 0.052 (0.030) | – | – | 23.400^*^ | <0.001^*^ |
| **Sample 8** | 0.06 ± 0.01 (0.006) | 0.51^a^ ± 0.259 (0.150) | 0.18 ± 0.053 (0.031) | 0.19 ± 0.058 (0.034) | 0.22 ± 0.046 (0.026) | 0.29 ± 0.127 (0.073) | 4.534^*^ | 0.015^*^ |
| **Sample 9** | 0.06 ± 0.01 (0.006) | 0.19^a^ ± 0.031 (0.018) | 0.09^b^ ± 0.010 (0.006) | 0.13^ab^ ±0.013 (0.007) | 0.15^a^ ± 0.025 (0.015) | 0.17^a^ ± 0.016 (0.009) | 20.325^*^ | <0.001^*^ |
| **Sample 10** | 0.06 ± 0.01 (0.006) | 0.19^a^ ± 0.038 (0.022) | 0.09^b^ ± 0.006 (0.004) | 0.10^b^ ± 0.004 (0.002) | 0.11^ab^ ±0.002 (0.001) | 0.13^ab^ ±0.010 (0.006) | 19.649^*^ | <0.001^*^ |
| **Sample 11** | 0.06 ± 0.01 (0.006) | 0.64^a^ ± 0.073 (0.042) | 0.32^ab^ ±0.070 (0.041) | 0.46^ab^ ±0.026 (0.015) | 0.58^a^ ± 0.020 (0.012) | 0.68^a^ ± 0.057 (0.033) | 66.836^*^ | <0.001^*^ |
| **Sample 12** | 0.06 ± 0.01 (0.006) | 0.45^a^ ± 0.034 (0.020) | 0.22^ab^ ±0.025 (0.014) | 0.27^ab^ ±0.012 (0.007) | 0.29^ab^ ±0.002 (0.001) | 0.36^ab^ ±0.027 (0.015) | 115.901^*^ | <0.001^*^ |
| **Sample 13** | 0.06 ± 0.01 (0.006) | 0.36^a^ ± 0.044 (0.026) | 0.20^ab^ ±0.015 (0.008) | 0.27^ab^ ±0.020 (0.011) | 0.33^a^ ± 0.026 (0.015) | 0.34^a^ ± 0.038 (0.022) | 48.111^*^ | 0.001^*^ |
| **Sample 14** | 0.06 ± 0.01 (0.006) | 0.74^a^ ± 0.008 (0.005) | 0.28^ab^ ±0.010 (0.006) | 0.35^ab^ ±0.028 (0.016) | 0.40^ab^ ±0.008 (0.005) | 0.42^ab^± 0.006 (0.003) | 786.850^*^ | <0.001^*^ |
| **Sample 15** | 0.06 ± 0.01 (0.006) | 2.11^a^ ± 0.597 (0.345) | 0.56^b^ ± 0.060 (0.035) | 0.82^ab^ ±0.073 (0.042) | 1.13^ab^ ±0.235 (0.136) | 1.71^a^ ± 0.090 (0.052) | 23.742^*^ | <0.001^*^ |
| **Sample 16** | 0.06 ± 0.01 (0.006) | 0.25 ± 0.008 (0.005) | 0.08 ± 0.005 (0.003) | 0.69^a^ ± 0.509 (0.294) | 0.11 ± 0.009 (0.005) | 0.19 ± 0.007 (0.004) | 3.863^*^ | 0.026^*^ |
| **Sample 17** | 0.06 ± 0.01 (0.006) | 0.82^a^ ± 0.263 (0.152) | 0.27^b^ ± 0.015 (0.009) | 0.34^b^ ± 0.031 (0.018) | 0.44^ab^ ±0.020 (0.012) | 0.45^ab^ ±0.064 (0.037) | 15.043^*^ | <0.001^*^ |
| **Sample 18** | 0.06 ± 0.01 (0.006) | 0.12^a^ ± 0.010 (0.006) | 0.08 ± 0.006 (0.003) | 0.10^a^ ± 0.008 (0.004) | 0.11^a^ ± 0.002 (0.001) | 0.13^a^ ± 0.017 (0.010) | 21.792^*^ | <0.001^*^ |
| **Sample 19** | 0.06 ± 0.01 (0.006) | 0.95^a^ ± 0.206 (0.119) | 0.27^b^ ± 0.020 (0.012) | 0.36^ab^ ±0.015 (0.009) | 0.66^a^ ± 0.049 (0.028) | 0.82^a^ ± 0.166 (0.096) | 29.167^*^ | <0.001^*^ |
| **Sample 20** | 0.06 ± 0.01 (0.006) | 0.49^a^ ± 0.078 (0.045) | 0.28^ab^ ±0.041 (0.023) | 0.28^ab^ ±0.011 (0.006) | 0.32^ab^ ±0.011 (0.006) | 0.36^ab^ ±0.007 (0.004) | 43.909^*^ | <0.001^*^ |
| **ATCC13637** | 0.06 ± 0.01 (0.006) | 0.27^a^ ± 0.056 (0.032) | 0.08^b^ ± 0.004 (0.002) | 0.10^b^ ± 0.018 (0.010) | 0.12^b^ ± 0.033 (0.019) | 0.20^a^ ± 0.083 (0.048) | 9.942 | 0.001^*^ |

3 replica for each group Data was expressed using Mean ± SD. (SE)

SD: **Standard deviation** SE: **Standard error of mean**

**F**: **F for One way ANOVA test**, Pairwise comparison bet. each 2 groups was done using **Post Hoc Test (Tukey)**

p: p value for comparing between the studied groups

*: Statistically significant at p ≤ 0.05

**a: Significant with Negative control**

**b: Significant with Positive control**
